# Introgression of a major QTL from an inferior into a superior population using genomic selection

**DOI:** 10.1186/1297-9686-41-38

**Published:** 2009-07-27

**Authors:** Jørgen Ødegård, Anna K Sonesson, M Hossein Yazdi, Theo HE Meuwissen

**Affiliations:** 1 Nofima Marine, PO Box 5010 NO-1432, Ås, Norway; 2 Department of Animal and Aquacultural Sciences, Norwegian University of Life Sciences, PO Box 5003, NO-1432, Ås, Norway

## Abstract

**Background:**

Selection schemes aiming at introgressing genetic material from a donor into a recipient line may be performed by backcross-breeding programs combined with selection to preserve the favourable characteristics of the donor population. This stochastic simulation study investigated whether genomic selection can be effective in preserving a major quantitative trait locus (QTL) allele from a donor line during the backcrossing phase.

**Methods:**

In a simulation study, two fish populations were generated: a recipient line selected for a production trait and a donor line characterized by an enhanced level of disease resistance. Both traits were polygenic, but one major QTL affecting disease resistance was segregating only within the donor line. Backcrossing was combined with three types of selection (for total merit index) among the crossbred individuals: classical selection, genomic selection using genome-wide dense marker maps, and gene-assisted genomic selection. It was assumed that production could be observed directly on the selection candidates, while disease resistance had to be inferred from tested sibs of the selection candidates.

**Results:**

Classical selection was inefficient in preserving the target QTL through the backcrossing phase. In contrast, genomic selection (without specific knowledge of the target QTL) was usually effective in preserving the target QTL, and had higher genetic response to selection, especially for disease resistance. Compared with pure genomic selection, gene-assisted selection had an advantage with respect to disease resistance (28–40% increase in genetic gain) and acted as an extra precaution against loss of the target QTL. However, for total merit index the advantage of gene-assisted genomic selection over genomic selection was lower (4–5% increase in genetic gain).

**Conclusion:**

Substantial differences between introgression programs using classical and genomic selection were observed, and the former was generally inferior with respect to both genetic gain and the ability to preserve the target QTL. Combining genomic selection with gene-assisted selection for the target QTL acted as an extra precaution against loss of the target QTL and gave additional genetic gain for disease resistance. However, the effect on total merit index was limited.

## Background

In domesticated populations, specific traits of interest may be improved through introgression of genes from a donor line favourable with respect to a trait of interest (*e.g*., a wild population resistant to a specific disease). However, the donor line is often inferior with respect to other traits included in the breeding objective of the recipient line (*e.g*., production), which hampers crossbreeding. Introgression schemes may be carried out through a backcross breeding program aimed at introgressing a single gene from the donor line into the genomic background of a recipient line, where molecular markers can be used to assess the presence of the introgressed gene [1]. Successful marker-assisted introgression programs have been conducted, using markers linked to known QTL positions [2,3]. An alternative strategy to traditional introgression schemes is to combine introgression and QTL detection into a single step [4]. An even simpler approach is to combine introgression with a genomic selection program [5], where individuals are selected based on estimated marker effects distributed over the entire genome. Hence, within-strain selection for desired alleles from both lines may be initiated directly on the F1 crossbreds avoiding loss of time on initial QTL detection studies.

In a previous simulation study [6], genetic material was introgressed from a donor line (inferior with respect to production, but superior with respect to disease resistance), into a recipient line (superior with respect to production, but inferior with respect to disease resistance), assuming that both traits were controlled by many QTL. The results indicated that, compared with pure breeding, an introgression program using genomic selection produces a more resistant and economically competitive crossbred population within relatively few generations [3-5]. Therefore, introgression combined with genomic selection was suggested as a tool for introgressing genetic material from inferior donor lines into recipient populations. The current study is an extension of the previous study, taking a major QTL into consideration.

Typical introgression programs, aim at introgressing one or more alleles of interest from the donor line, while simultaneously reducing the amount of donor DNA to a minimum. Here, genomic selection will not necessarily reduce the amount of donor DNA, but should lead to an increase of the frequency of favourable alleles, irrespective of their origin [6]. In simulated introgression schemes, Groen and Smith [7] have concluded that selection for genomic similarity to the recipient line is less efficient than selection for phenotype. In the current study, individuals are selected based on total genetic value, summed over all markers (irrespective of origin). Hence, any favourable allele from the donor line may be introgressed. Introgression of major QTL alleles is therefore likely, although not assured. This strategy is particularly relevant when (major) QTL are not known prior to selection.

The aim of this study was to investigate whether genomic selection methods [5] can be used to introgress a major QTL allele through a backcrossing program in a situation where both the location and the effect of the QTL alleles are unknown, and when the trait is also affected by numerous minor genes. The genomic selection schemes was compared with classical selection schemes without the use of any genomic information and schemes using genomic selection for minor QTL combined with gene-assisted selection for the major QTL. The latter alternative can be seen as a best-case scenario for introgression of specific QTL alleles of interest.

## Methods

The selection experiment was designed to use genetic resources from two partially separated populations. Generally, two fish populations were simulated as in Ødegård *et al*. [6]. The common base population was generated and mated randomly with replacement for 10,000 generations (effective population size N_e _= 1000), subsequently the base population was randomly split into two equally sized (N_e _= 1000) subpopulations, which were kept separate for the following 250 generations. Finally, for 10 generations, one population (recipient line) was phenotypically selected for a production trait (PT), with a heritability of 0.1, by selecting the top 10% of males and top 10% of females. The other population (donor line) was randomly selected with replacement (random 10% of the males and females). The purpose of the separation period was to generate two partially differentiated populations that were at different genetic levels for important traits. A single major QTL for disease resistance (DR) was assumed to segregate only within the donor line. Randomly selected sires and dams from the resulting two lines were then used as parents for two lines; one purebred recipient line selected for PT only (PRL = production line) and an F1 cross in the following selection experiment over five generations (S1 to S5). Different replicates were simulated separately, but the initial generation (S0) was identical for all scenarios within each replicate.

### Selection

Two selection strategies were used for generations S1 – S5: 1) Pure breeding of the recipient line for backcrossing for a breeding objective including only one production trait (PT), *i.e*., a production line (PRL), which represents an external commercial population included in a classical selection program for improved production, and 2) an introgression strategy by creating F1 crossbreds of recipient and donor lines, followed by repeated backcrossing to the PRL, *i.e*., a BACKCROSS line. Backcrossing was done only to the extent that females from the PRL were superior to females recruited from within the crossbred population based on their EBV for total merit index (TMI), *i.e*., females were selected among all candidates in both populations, while males were selected within the crossbred population only.

For generations S0 to S5, all lines were kept at a constant size of 1000 breeding candidates within each line. For the parents of S0, random selection and mating using sampling with replacement was applied, while, for the later generations, truncation selection based on predicted EBV was used (50 sires and 50 dams per line). The selected sires and dams were randomly mated (using sampling with replacement) to create 50 full-sib families with 20 offspring each to form selection candidates for the next generation for each line. Additionally, all 50 families within the BACKCROSS line each produced 20 offspring, which were used in sib-testing for DR.

### Genome structure

The genome structure in this simulation was identical to that of Ødegård *et al*. [6], *i.e*., with 10 diploid 100 cM chromosomes assuming the Haldane mapping function and a Mendelian inheritance of all loci. For each chromosome, 500 marker loci were assumed, as well as 100 QTL per trait (PT and DR). Markers and QTL loci were randomly spaced throughout each chromosome. Rates of mutations (per allele and meiosis for each generation) for marker and QTL alleles were 0.0001 and 0.00001, respectively. Mutation rates at markers were increased to ensure that most markers were segregating. All mutations generated new alleles, and thus, all loci were potentially multi-allelic. The QTL allelic effects were assumed to be additive, and were sampled from a gamma distribution (shape and scale parameters of 0.40 and 0.13, respectively). Since this distribution only produces positive values, each QTL allelic effect had a 50% probability of being switched to a negative value. No pleiotropy was assumed, implying zero genetic covariance between the traits before selection. At generation t = 10,250 (10,000 + 250), QTL effects of both traits were scaled to achieve identical background genetic standard deviations (= 1.0) for both traits within the recipient line for all replicates (before selection). Scaling of QTL effects was identical for all individuals, irrespective of population.

At generation t = 10,260 (10,000 + 250 + 10), ~80% of the marker loci and ~15% of the QTL were segregating within each subpopulation. Linkage disequilibrium between adjacent markers was calculated as the standardized chi-square, *χ*^2^' [8,9]. Within each base population, average calculated and expected [10] LD for adjacent markers (expectation based on actual distance) were both 0.2.

At generation S0 of the selection experiment, genomes of all individuals were scanned to identify bi-allelic QTL affecting DR, where one of the alleles happened to be fixed in the recipient line, but an alternative allele existed in the donor line. The QTL displaying the largest difference in allele frequencies between the lines was assumed to be a major QTL. The favourable allele (the allele absent in the recipient line) was given a genotypic value [11] of a = 2.0 (twice the background genetic standard deviation), while the genotypic value of the alternative allele was a = -2.0.

### Data

As in the study by Ødegård *et al*[6]., the true breeding value of an individual was defined as the sum of QTL allelic effects for the individual across all 1000 QTL loci for each trait. Phenotypes of both traits were produced by adding normally distributed error terms, sampled from *N*(0, ), to the true breeding values of each individual. Heritabilities are presented as the following:  where  is the (background) additive genetic variance (= 1.0 for both traits) within the original recipient line (not segregating for the major QTL), and  is the residual variance (= 9.0 for both traits). The resulting background heritability (not accounting for the major QTL) was therefore 0.10 for both PT and DR. It was assumed that all individuals within the BACKCROSS line were genotyped for the available 5000 marker loci. The PT was recorded on all selection candidates (1000 individuals per line and generation), while disease resistance (DR) was recorded on full-sibs of the selection candidates, using a challenge-test type of design (1000 individuals per generation for the BACKCROSS line). For the PRL, only the average genetic level of DR was assumed to be available. Individuals challenge-tested for DR were not considered as selection candidates.

### Breeding value estimation

For comparison purposes, genomic (GBLUP), gene-assisted genomic (GasGBLUP) and classical (CBLUP) EBV were produced and used as selection criteria. The GBLUP were estimated as in Ødegård *et al*. [6] using the BLUP estimation procedure of marker effects [5]. Marker by base population effects were estimated, *i.e*., all markers were traced back to their original populations. For GasGBLUP, the same method was used, but the effect of the major QTL was assumed known in the breeding value estimation. The CBLUP were estimated using classical BLUP [12]. The CBLUP of DR for selection candidates was calculated based on sib and pedigree data (including phenotypes back to generation S0). The GBLUP and GasGBLUP values were scaled by a factor *b *, in order to make them directly comparable to CBLUP values. All breeding values and the factor *b *were re-estimated for each generation (the latter was based on all individuals with genomic EBV across generations).

### Scenarios

Backcrossing schemes were conducted for the different selection criteria (using CBLUP, GBLUP and GasGBLUP). Two sets of economic weights were used; either 100% (2:1) (Scenario 1) or 50% (3:2) (Scenario 2) higher relative weight on PT compared with DR (Table 1). In the crossbred population, selection was for total merit index (TMI), with the selection criteria defined as the sum of predicted breeding values for both traits, multiplied by their corresponding economic weights. For all settings, the PRL was selected for PT using only the CBLUP selection criterion.

**Table 1:** Description of the selection schemes

**Scenario**	**Population structure**	**Selection criteria**	**EW PT**	**EW DR**
1	PRL	CBLUP	1	0
	BACKCROSS	CBLUP	2	1
	BACKCROSS	GBLUP	2	1
	BACKCROSS	GasGBLUP	2	1

2	PRL	CBLUP	1	0
	BACKCROSS	CBLUP	3	2
	BACKCROSS	GBLUP	3	2
	BACKCROSS	GasGBLUP	3	2

PT = production trait, DR = disease resistance, EW = relative economic weight, assuming equal genetic variance for the two traits

### Calculation of summary statistics

A total of 50 replicates were produced for each scenario and selection scheme. Within each replicate, the average frequency of the favourable major QTL allele and the average level of true breeding values (DR, PT and TMI) were calculated, and subsequently averaged over all 50 replicates. Genetic gains for PT, DR and TMI were calculated as average differences in true genetic level from generation S0 to S5. For comparison purposes, the expected frequency of the target QTL given no selection (but identical amounts of backcrossing) was calculated as the expected genetic contribution of the donor population (based on pedigree) multiplied with the original frequency of the favourable target allele in the donor population.

## Results

At generation S0, the average genetic differences (in background genetic standard deviations) between the recipient (PRL) and the F1 crossbred populations were 2.5 and -0.4 for PT and DR, respectively (difference in DR mainly due to the major QTL). Consequently, TMI differed for the two populations. Assuming 100% higher economic weight for PT (economic weights 2:1), the average difference in TMI was 2.1 genetic standard deviations, and 1.9 genetic standard deviations, assuming 50% higher economic weight for PT (economic weights 3:2). The initial frequency of the favourable major QTL allele was 0% in the PRL (recipient) line and, on average, 23% in the F1 BACKCROSS population.

### Scenario 1: ratio of economic weights for PT and DR 2:1

**Figure 1:**
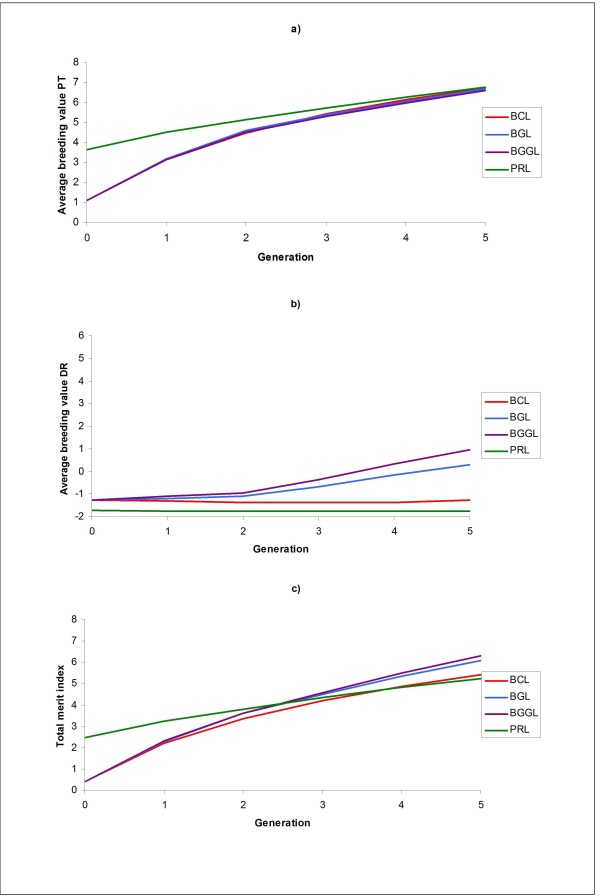
**Genetic levels for a) production trait (PT), b) disease resistance (DR) and c) total merit index (TMI) by generation for the different selection schemes under scenario 1; the selection schemes presented are; PRL, BACKCROSS CBLUP (BCL), BACKCROSS GBLUP (BGL) and BACKCROSS gene-assisted GBLUP (BGGL)**.

Genetic levels by generation for PT, DR and TMI are shown in Figure (1a, b and 1c, respectively) and genetic gains (from S0 to S5) are shown in Table 2. As in Ødegård *et al*. [6], genomic selection was in general favourable compared with classical selection. With respect to DR (the trait affected by the major QTL), there were substantial differences in genetic gain between the alternatives using classical selection (CBLUP) and those using genomic selection (GBLUP and GasGBLUP). As a result of repeated backcrossing with the PRL, no significant genetic gain for DR was achieved through CBLUP. However, substantial genetic gain was achieved when using genomic selection, both as a result of the more efficient within-line selection and as a result of less sustained backcrossing with the PRL. As expected, genetic gain in DR was the highest with GasGBLUP (increase by 40%, compared with GBLUP). The selection methods differed much less with respect to genetic gain for PT, *i.e*., selection for improved PT was about as efficient using genomic or classical selection (-1% and -2% for GBLUP and GasGBLUP, respectively) despite more backcrossing with PRL under classical selection. Consequently, genetic gain in TMI was higher for genomic than for classical selection (13 and 18% increase for GBLUP and GasGBLUP, respectively). The relatively limited advantage of GasBLUP over GBLUP with respect to genetic gain in TMI (4%) can be explained by the high relative economic weight of PT.

**Table 2:** Average genetic gains from generation S0 to S5 of the different selection schemes for production trait (PT), disease resistance (DR) and total merit index (TMI).

**Scenario**	**Population structure**	**Selection criteria**	**Genetic gain PT**	**Genetic gain DR**	**Genetic gain TMI**
**1**	PRL	CBLUP	3.12 (0.10)	-0.04 (0.07)	2.78 (0.09)
	BACKCROSS	CBLUP	5.61 (0.17)	0.01 (0.11)	5.02 (0.16)
	BACKCROSS	GBLUP	5.56 (0.19)	1.59 (0.13)	5.69 (0.14)
	BACKCROSS	GasGBLUP	5.49 (0.18)	2.24 (0.12)	5.91 (0.14)

**2**	PRL	CBLUP	3.12 (0.10)	-0.04 ((0.07)	2.78 (0.09)
	BACKCROSS	CBLUP	5.44 (0.17)	0.32 (0.11)	4.71 (0.13)
	BACKCROSS	GBLUP	5.15 (0.19)	2.14 (0.12)	5.48 (0.13)
	BACKCROSS	GasGBLUP	5.09 (0.17)	2.73 (0.09)	5.75 (0.13)

All genetic gains are measured in Background genetic standard deviations with standard errors given in parentheses

PT = production trait, DR = disease resistance, EW = relative economic weight, assuming equal genetic variance for the two traits

**Figure 2:**
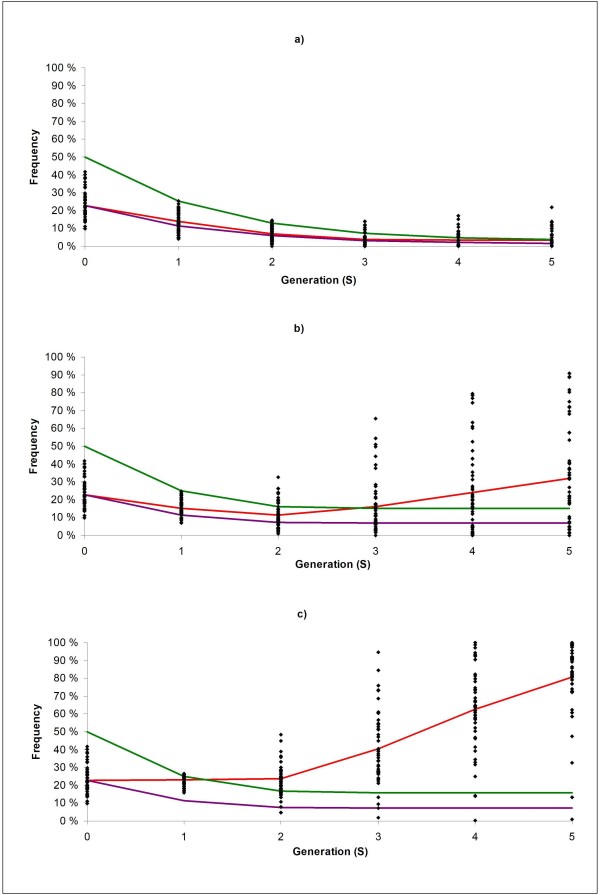
**Allele frequencies of the favourable allele of the major QTL for the BACKCROSS selection scheme under a) classical (CBLUP), b) genomic (GBLUP), and c) gene-assisted genomic (GasGBLUP) selection in Scenario 1**. Single dots represent individual replicates, red line average observed frequencies of the target QTL, purple line expected frequencies if the target QTL is not subject to selection and green line expected frequencies of "neutral" donor alleles.

The frequencies of the favourable major QTL allele by generation are shown in Figure 2 (single replicates and average level). In the introgression schemes using classical selection (CBLUP), backcrossing with the PRL line was continued throughout the entire selection experiment, and average expected frequency of "neutral" donor alleles (based on pedigree) was therefore as low as 4% at generation S5 (Figure 2a). Consequently, average frequency of the favourable major QTL allele also dropped from 23% at generation S0 to 4% in generation S5, and ended up as lost in 27 out of 50 replicates (Figure 2a). For the introgression schemes using genomic selection (GBLUP/GasGBLUP), backcrossing with the PRL mainly occurred up to generation S2, and the expected frequency of "neutral" donor alleles in the GBLUP/GasGBLUP alternatives thus stabilized at, respectively, 15 and 16% towards the end of the selection experiment (Figure 2b and 2c). Using GBLUP, the average frequency of the favourable allele for the major QTL dropped from 23% at generation S0 to 11% at generation S2 (Figure 2b), as a result of backcrossing with PRL. Using GasGBLUP, the frequency of the favourable major QTL allele was stable for S0 to S2 (Figure 2c). However, as a result of genomic/gene-assisted selection within the crossbred population, the average frequency of the major QTL increased from S2 and onwards for both GBLUP and GasGBLUP (to respectively 32% and 81% in generation S5). The favourable QTL allele was never lost using GasGBLUP, but it was lost in 6 out of 50 replicates using GBLUP.

### Scenario 2: ratio of economic weights for PT and DR 3:2

**Figure 3:**
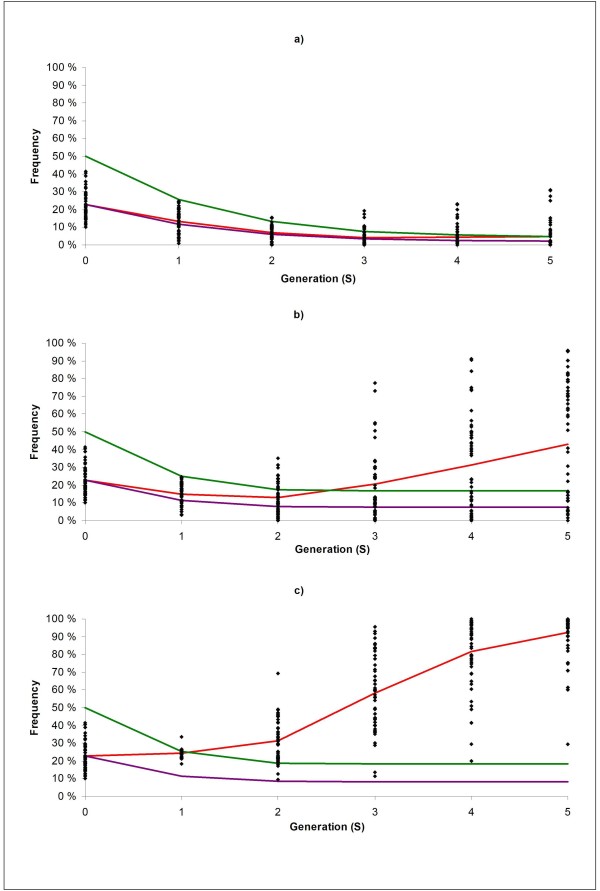
**Allele frequencies of the favourable allele of the major QTL for the BACKCROSS selection scheme under a) classical (CBLUP), b) genomic (GBLUP), and c) gene-assisted genomic (GasGBLUP) selection in Scenario 2**. Single dots represent individual replicates, red line average observed frequencies of the target QTL, purple line expected frequencies if the target QTL is not subject to selection and green line expected frequencies of "neutral" donor alleles.

Genetic gains (from S0 to S5) are shown in Table 2. Generally, ranking of selection schemes was similar to that in Scenario 1, with the largest differences in genetic gain observed for DR, and the smaller differences in genetic gain observed for PT. Again, genomic selection increased genetic gain for TMI compared with classical selection (16 and 22% for GBLUP and GasGBLUP, respectively). Classical selection led to substantial backcrossing, and the expected amount of "neutral" donor alleles was therefore as low as 5% in generation S5 (Figure 3a), and had a limited effect on the frequency of the favourable major QTL, reaching an average frequency of 5% in generation S5, while being lost in 29 out of 50 replicates (Figure 3a).

Compared with GBLUP, GasGBLUP selection was superior with respect to genetic gain in DR (28%), slightly inferior with respect to genetic gain in PT (-1%) and slightly superior with respect to genetic gain in TMI (5%). Again, backcrossing with the PRL mainly occurred up to generation S2 in both GBLUP and GasGBLUP selection schemes, and the expected frequency of "neutral" donor alleles stabilized at 17% and 18%, respectively (Figure 3b and 3c). For the GBLUP selection scheme, the frequency of the favourable major QTL allele followed the same pattern as in the scenario above (Figure 2b), although the initial drop (from S0 to S2) in the average frequency of the favourable major QTL allele was smaller (from 23 in S0 to 13% in S2), as expected through reduced backcrossing. For the GasGBLUP selection scheme (Figure 3c), the frequency of the favourable major QTL allele increased throughout the entire selection experiment, but most markedly after generation S2, where backcrossing had essentially ceased. The average end-frequency of the favourable major QTL allele (at S5) was 43% and 93%, for GBLUP and GasGBLUP schemes, respectively. Again, the favourable allele was never lost using GasGBLUP, but it was lost in 5 out of 50 replicates for GBLUP.

## Discussion

The results of this study clearly show that genomic selection can to a large extent identify relevant DNA segments for both traits and use this information in selection, while classical selection has a limited effect on alleles affecting DR, even for a QTL of very large effect. Consequently, backcrossing programs using GBLUP selection are far more efficient in preserving and increasing frequencies of such alleles, while for CBLUP selection programs these alleles may easily be lost during the backcrossing process. The advantage of genomic over classical selection with respect to a major QTL for DR may be explained by two main factors: 1) generally higher accuracy of selection for GBLUP compared with CBLUP [5] and 2) for a trait solely recorded on sibs of selection candidates, classical selection can only distinguish between families, while genomic selection may distinguish between single individuals of different genotypes within families segregating for the major QTL.

With respect to the major QTL, the most efficient selection schemes were those combining genomic selection with gene-assisted selection for the target QTL. However, the relative advantage of GasBLUP relative to GBLUP selection was rather small with respect to genetic gain in TMI (4–5%). In GBLUP selection, the favourable allele was lost in a few replicates (5–6 out of 50), which never happened using GasGBLUP. Therefore, the results of this study indicate that the main advantage of GasGBLUP compared with GBLUP selection is that it acts as a precaution against the loss of the favourable major QTL allele during the early backcrossing phase. This is of special importance if the allele of interest has a low frequency within the donor population, and when the target QTL is in unfavourable LD with other loci, since selection on the target QTL would be detrimental for background genetic effects. However, because of the latter, rapid introgression of the major QTL is not necessarily a substantial advantage. As long as the favourable allele of the target QTL is preserved, it would most likely approach fixation in the long run. Further, according to a study by Gibson [13], less intense selection on a major QTL is likely to increase the long-term genetic gain, implying that GBLUP selection is expected to be superior over GasGBLUP selection in the long run, except for replicates where the favourable QTL alleles were lost during the initial backcrossing process. However, in Gibson's study, unfavourable LD between the major QTL and the background genetic effects were generated as a result of selection, while in our case, such unfavourable relationships are present already at the start of the selection experiment as a result of crossing the two base populations, *i.e*., the positive QTL allele is located on a donor chromosome segment that is more likely to contain negative alleles with respect to PT. Thus, in the current introgression scheme, substantial weight on the target QTL is needed in order to avoid its loss, whereas in Gibson's single population, risk of loss is low even if the major QTL receives little weight.

In this study, the genotypic value of the major QTL implies that this locus explains 2/3 of the total genetic variance in DR within a population having an allele frequency of 50% (assuming Hardy-Weinberg equilibrium). The advantage of efficient introgression of the favourable allele will increase with the effect of the allele and with the economic weight of DR. However, for all selection methods, the efficiency of selection with respect to the target QTL will also increase with the same factors. Preliminary analyses using Scenario 2, showed that for a doubled genotypic value of the target QTL, GasGBLUP selection only slightly increased genetic gain for DR (7%) and hardly increased genetic gain in TMI (1%), compared with GBLUP selection (results not shown). Hence, the relative advantage of gene-assisted selection is seemingly lower for QTL of very large effect, since the favourable allele would be preserved and effectively selected for even without gene-assisted selection.

The GasGBLUP schemes require that knowledge on both the exact position of the target QTL and its effect exists prior to the selection experiment, in addition to a dense marker map including segregating marker loci for both populations. If this knowledge is readily available prior to the experiment, the GasGBLUP method is naturally preferred. This can be considered a best-case scenario, but in practical breeding schemes, these assumptions would often be unrealistic. The GBLUP selection schemes do not require prior knowledge about the target QTL, and are therefore much easier to implement. An alternative approach would be to do simultaneous QTL detection and introgression as described in Yazdi *et al*. [4]. However, the latter approach would be less efficient when applied to complex traits affected by numerous QTL of varying effects, while genomic selection is well suited to handle this situation.

Due to computing time considerations, the BLUP method of genomic selection was used. This method assumes homogeneous genetic variance at all marker loci [5]. However, more advanced methods of genomic selection are also available, *i.e*., the so-called BayesA and BayesB methods [5]. Here, the genetic variance explained by each marker locus is evaluated and different weights are thereby given to different genomic regions in the EBV calculation. Thus, these more advanced methods are expected to be more effective in selecting QTL of major importance, and the results of the current study should therefore be considered as conservative. In a recent study [14], where analyses were based on simulated crossbred populations using the BayesB method, it was concluded that fitting population-specific marker effects may not be necessary, especially for high marker densities. However, using the BLUP method, as in the current study, all markers are assumed to have effects of equal magnitude, not only the markers in closest association with QTL (as in BayesB). Hence, fitting population-specific markers may be of more importance, since QTL effects are likely to be attributed to a broader distribution of markers.

In previous stochastic simulation or deterministic studies of introgression programs targeting a major QTL, donor and recipient lines are often assumed to be unrelated and fixed for alternative target QTL alleles [1,15,16]. For livestock and farmed fish, these assumptions are usually unrealistic, and provide idealized conditions for the introgression programs. In the current study, the donor and recipient lines were assumed to be only partially differentiated, and the target QTL was chosen among loci that happened to be segregating only within the donor line (since this would make introgression necessary). Hence, introgression of the major QTL would not be guaranteed by crossing the recipient line with a small sub-sample of individuals from the donor line, and one would rarely have markers in perfect LD with the major QTL. However, some simplified assumptions were made, *i.e*., the assumption of no pleiotropy, implying zero genetic correlation (before selection) between disease resistance and the production trait (*e.g*., growth). The latter is not necessarily unrealistic, but the assumption of complete absence of pleiotropic effects on all loci is most likely over-simplified. Actually, numerous pleiotropic effects in both directions (favourable/unfavourable) may underlie a genetic correlation close to zero. However, under the current assumption, different QTL for the two traits will often be closely linked, which will have an effect that resembles pleiotropic QTL. The practical consequences of this assumption are thus likely to be limited. In our view, the current method of simulation and (GBLUP) analysis is rather realistic and conservative with respect to the prospects for introgression schemes in livestock and farmed fish populations.

## Conclusion

There were substantial differences between introgression programs using classical and genomic selection, with the first being generally inferior with respect to both genetic gain and the ability to preserve the target QTL. Combining genomic selection with gene-assisted selection for the target QTL acted as an extra precaution against loss of the target QTL and gave additional genetic gain for disease resistance. However, the effect on total merit index was limited compared with genomic selection without specific knowledge of the target QTL.

## Competing interests

The authors declare that they have no competing interests.

## Authors' contributions

JØ carried out the computer simulations and drafted the manuscript. MHY participated in software programming. AKS coordinated the project and helped to draft the manuscript. THEM participated in software programming and helped to draft the manuscript. All authors participated in the design of the study and read and approved the final manuscript.
